# Supplementary material for: Luteolin alleviates CUMS-induced depressive-like behavioral deficits in mice through blocking the JAK2/STAT3 pathway
Source: PLoS One. 2025 Oct 24;20(10):e0335057. doi: 10.1371/journal.pone.0335057 (PMC12551889; doi:10.1371/journal.pone.0335057)
Supplement: S1 File — (PDF) [file pone.0335057.s001.pdf]

actin

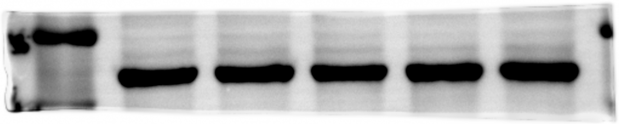

actin-marker

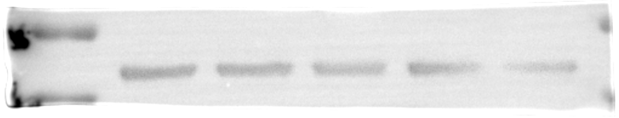

actin-sample

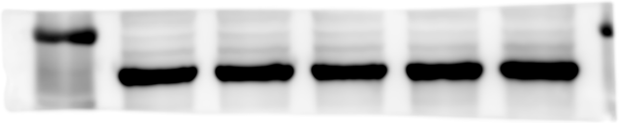

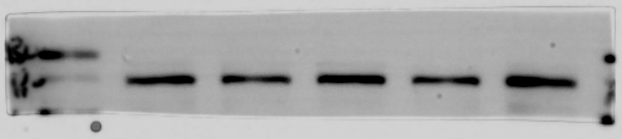

JAK2

JAK2-marker

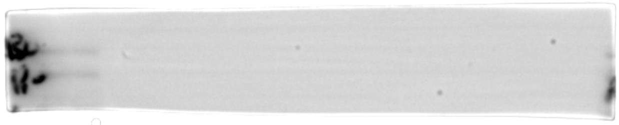

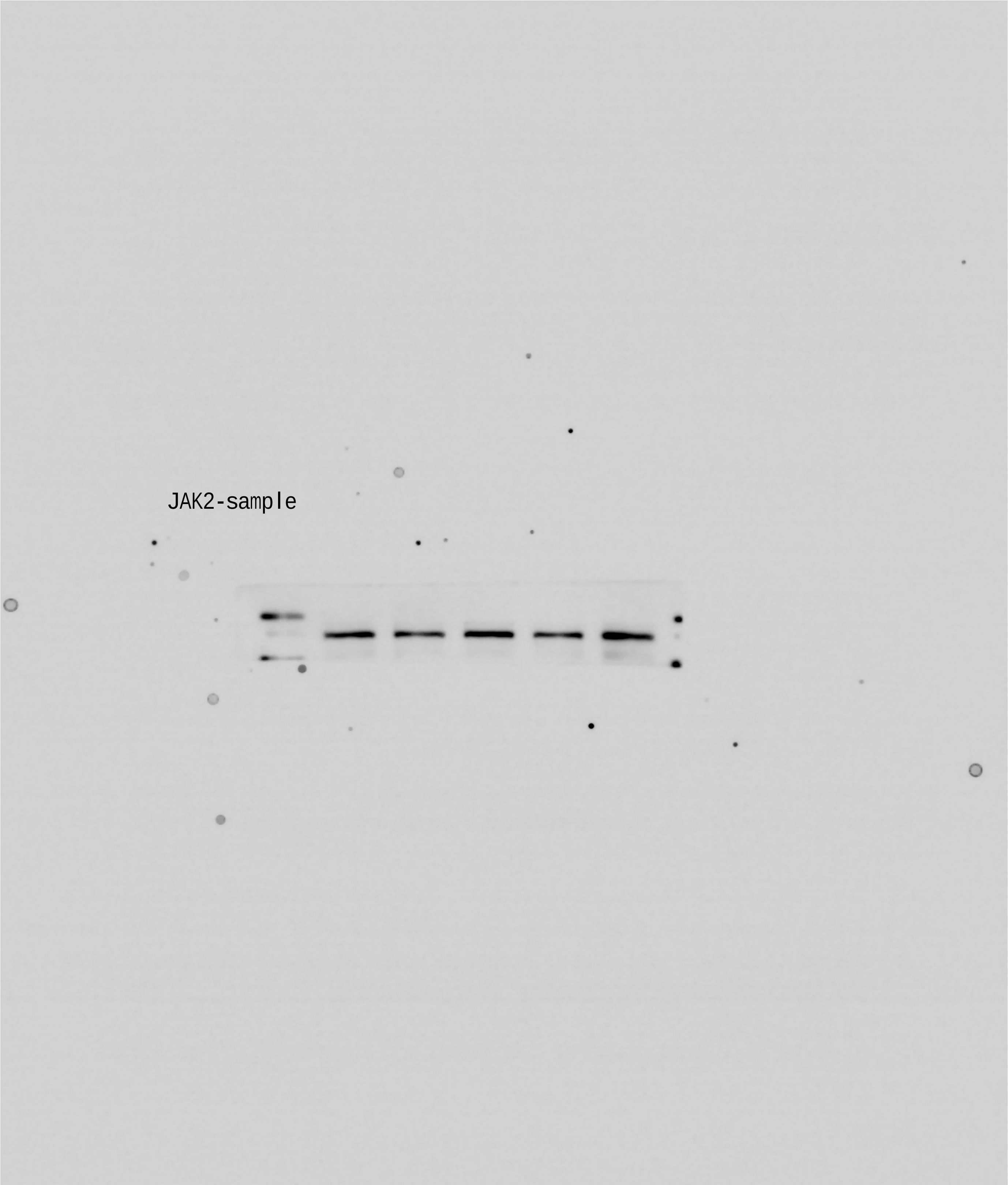
JAK2-sample

p-JAK2

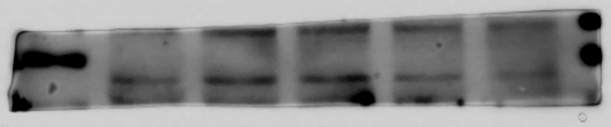

p-JAK2-marker

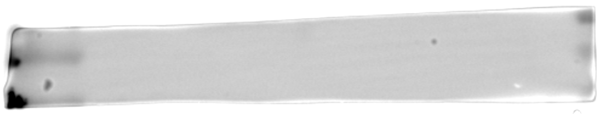

p-JAK2-sample

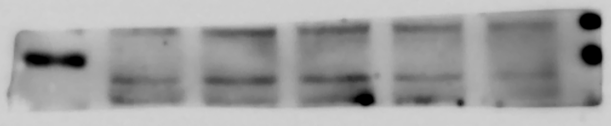

P-stat3

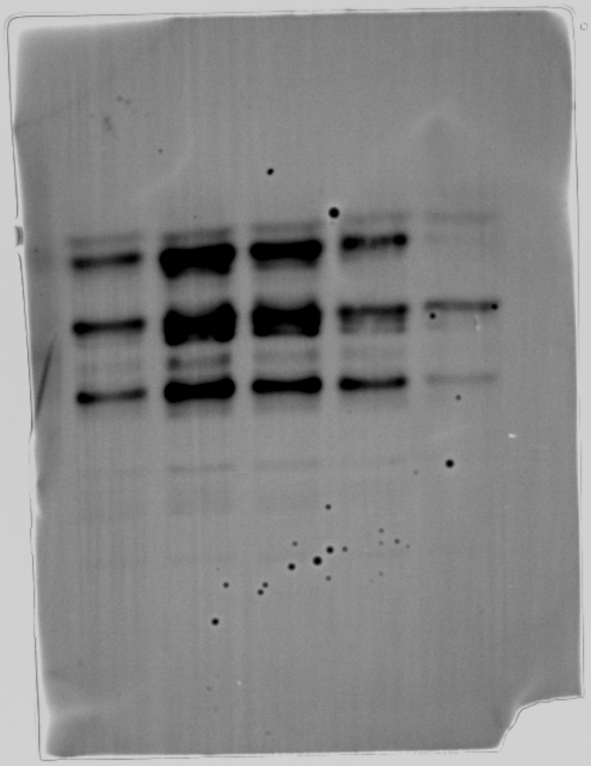

P-stat3-marker

O

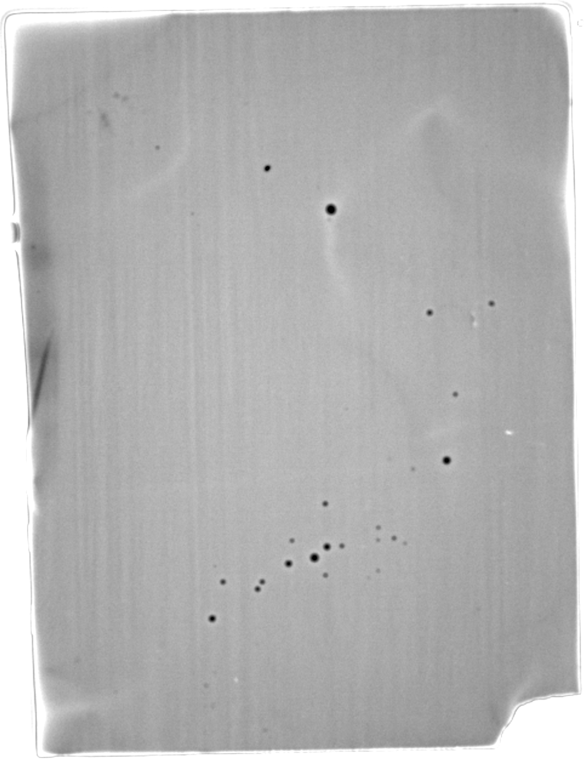

P-stat3-sample

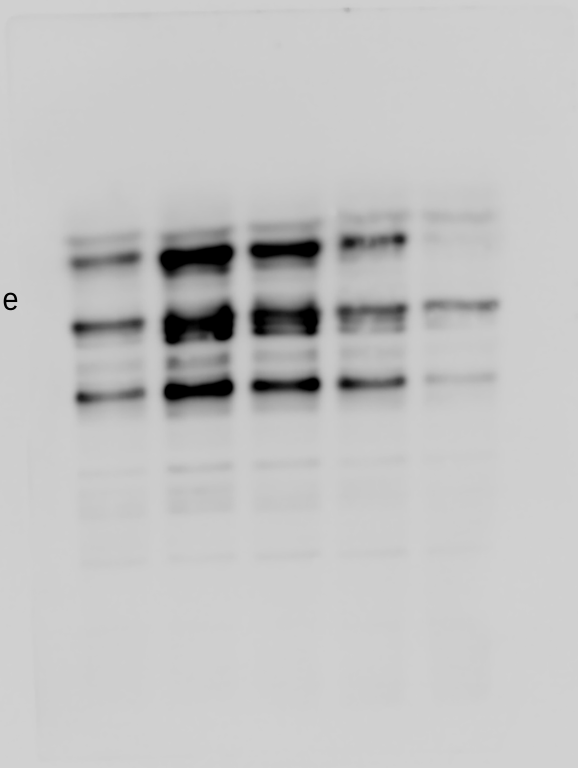

stat3

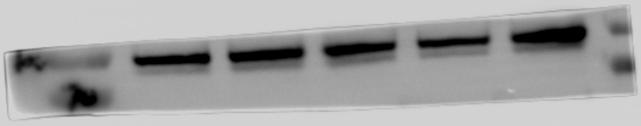

stat3-marker

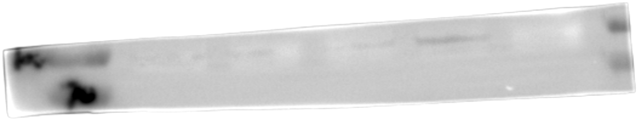

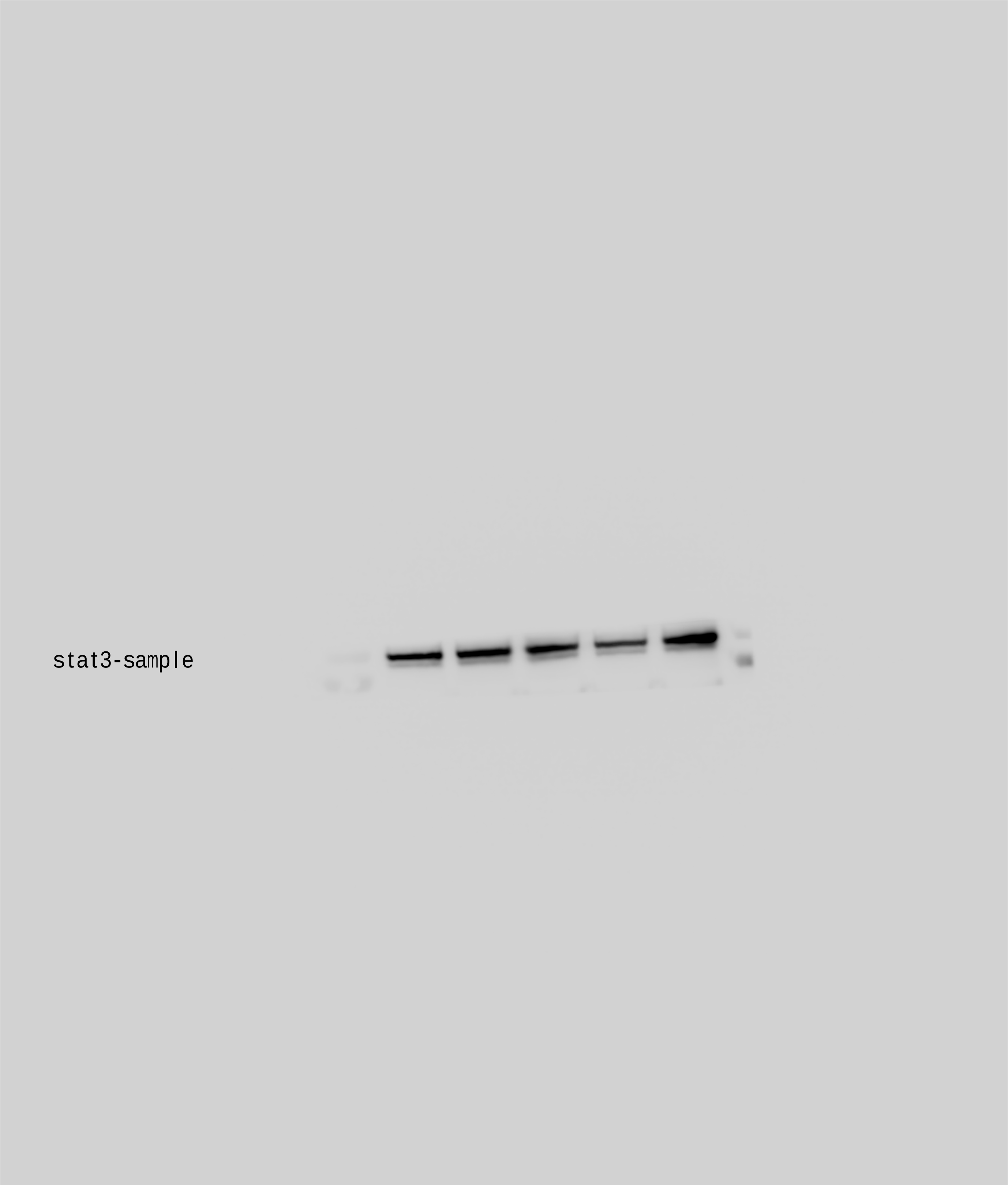
stat3-sample
