# Supplementary material for: Luteolin alleviates CUMS-induced depressive-like behavioral deficits in mice through blocking the JAK2/STAT3 pathway
Source: PLoS One. 2025 Oct 24;20(10):e0335057. doi: 10.1371/journal.pone.0335057 (PMC12551889; doi:10.1371/journal.pone.0335057)
Supplement: Attachment — Submitted filename: PONE-D-25-12815 - Review.docx [file pone.0335057.s002.docx]

The authors have shown that CUMS induces depressive-like behavior and increases p-JAK2, p-STAT3, and cytokine levels in mice. Furthermore, they’ve shown that luteolin reduces CUMS-induced effects on these parameters. My recommendation for this manuscript is to suggest major revisions for clarity and accurate representation of the statistics.

1. The statistical significance portrayed in the figures and legends does not match what is implied in the text. The figure captions state that the asterisks used on the LUT groups in the graphs show the significance compared to the control (no CUMS) group, but the data described in the text implies that the significance is in comparison to the CUMS group. Similarly, the figure captions state that the hashtag/number symbol was used to compare to the CUMS group, but that symbol is used on the CUMS group on the graph implying that the group was compared to itself. Either the statistics need to be described more clearly in the text or the figure captions just need to be updated.
2. Did the authors complete a power analysis to determine how many animals should be used in the behavioral tests? Typically, 10-12 animals would be used for these behavioral tests so eight seems low. If a power analysis was performed, that information should be included in the Materials and Methods section.
3. There is a discrepancy in the number of animals in the control group. The Materials and Methods section states n=6 but the results section states n=8.
4. Time spent in the central zone of the OFT was used as a measure of depressive-like behavioral deficits, and the results section states the OFT was used as a measure of motor abnormalities observed in depressed individuals. Citations should be included to show that the open field test has been used and validated in this way. The OFT is typically used to assess locomotor activity to ensure that a change in locomotor activity is not confounding the results of behavioral tests for depression, such as FST and TST, and not as a measure of depressive-like behavior itself.
5. The EPM is noted as being “commonly adopted to assess depressive-like behavioral deficits in rodents” and reference 7 is cited. Reference 7 does not use or state that EPM should be used as a measure of depressive-like behavior. Rather the EPM is used as a measure of anxiety. Because the results section described the EPM findings as anxiety, this may be a typo that can easily be fixed. If not, other references need to be included to show that the EPM has been used and validated in this way.
6. A positive control, *e.g.* fluoxetine, would be a welcome addition to this manuscript to validate the sensitivity of the paradigm and provide a comparison to established antidepressant treatments, however, it is not necessary for publication.
7. The results section mentions that “successful” CUMS mice were randomized into groups, but there is no mention of how “success” was defined. This criteria should be included in the Materials and Methods section.
8. The experimental set-up needs some clarification between the Materials and Methods section, the experimental workflow (Figure 1-1B), and the results section regarding the 2% ethanol solution (mentioned only in the results), 0.1 mL/kg of deionized water (mentioned only in the Materials and Methods), and DMSO (mentioned only in Figure 1-1B).
9. The results section should more transparently state the results of the low, medium, and high LUT groups. The results of the three LUT groups are often lumped together in the results section - stating that “LUT” had an effect – without mention of which dose(s) of LUT were significantly different in for each particular experiment.
10. The figure legends should state the type of tissue that was used for the experiments.
11. In section 2.2 of the results, it is stated in regard to the percentage of time spent in the open arms, “Although the other LUT doses showed a trend toward improvement, the differences were not statistically significant.” However, the graph shows that the LUT-M group was significant. The figure or text should be updated to reflect whichever information is correct. In addition, the p values have been stated, so mentioning a “trend” toward significance is irrelevant and unnecessary.
12. There is a typo in section 2.1 of the results section where “evtluate” is used instead of “evaluate”.
