# Supplementary material for: Luteolin alleviates CUMS-induced depressive-like behavioral deficits in mice through blocking the JAK2/STAT3 pathway
Source: PLoS One. 2025 Oct 24;20(10):e0335057. doi: 10.1371/journal.pone.0335057 (PMC12551889; doi:10.1371/journal.pone.0335057)
Supplement: Attachment — Submitted filename: Response to Reviewers.docx [file pone.0335057.s004.docx]

1.To comply with PLOS ONE submissions requirements, in your Methods section, please provide additional information regarding the experiments involving animals and ensure you have included details on (1) methods of sacrifice, (2) methods of anesthesia and/or analgesia, and (3) efforts to alleviate suffering.

**Author response 1**: We apologize for not making the necessary revisions in a timely manner. We have now supplemented the ethical details regarding animal experiments in Section 1.1 Animals. These details specifically include: euthanasia by cervical dislocation after deep anesthesia induced via intraperitoneal injection of 2% pentobarbital sodium, reducing the number of animals used through optimized experimental design, and minimizing the duration of stress during experiments to alleviate suffering as much as possible.

2. We note that your Data Availability Statement is currently as follows: All relevant data are within the manuscript and its Supporting Information files.

Please confirm at this time whether or not your submission contains all raw data required to replicate the results of your study. Authors must share the “minimal data set” for their submission. PLOS defines the minimal data set to consist of the data required to replicate all study findings reported in the article, as well as related metadata and methods (https://journals.plos.org/plosone/s/data-availability#loc-minimal-data-set-definition).

For example, authors should submit the following data:

- The values behind the means, standard deviations and other measures reported;

- The values used to build graphs;

- The points extracted from images for analysis.

Authors do not need to submit their entire data set if only a portion of the data was used in the reported study.

If your submission does not contain these data, please either upload them as Supporting Information files or deposit them to a stable, public repository and provide us with the relevant URLs, DOIs, or accession numbers. For a list of recommended repositories, please see https://journals.plos.org/plosone/s/recommended-repositories.

If there are ethical or legal restrictions on sharing a de-identified data set, please explain them in detail (e.g., data contain potentially sensitive information, data are owned by a third-party organization, etc.) and who has imposed them (e.g., an ethics committee). Please also provide contact information for a data access committee, ethics committee, or other institutional body to which data requests may be sent. If data are owned by a third party, please indicate how others may request data access.

**Author response 2**: We confirm that all data required to replicate the findings of this study have been deposited in a public repository. The DOI for the dataset is:10.6084/m9.figshare.30038803

3. PLOS ONE now requires that authors provide the original uncropped and unadjusted images underlying all blot or gel results reported in a submission’s figures or Supporting Information files. This policy and the journal’s other requirements for blot/gel reporting and figure preparation are described in detail at https://journals.plos.org/plosone/s/figures#loc-blot-and-gel-reporting-requirements and https://journals.plos.org/plosone/s/figures#loc-preparing-figures-from-image-files. When you submit your revised manuscript, please ensure that your figures adhere fully to these guidelines and provide the original underlying images for all blot or gel data reported in your submission. See the following link for instructions on providing the original image data: https://journals.plos.org/plosone/s/figures#loc-original-images-for-blots-and-gels.

In your cover letter, please note whether your blot/gel image data are in Supporting Information or posted at a public data repository, provide the repository URL if relevant, and provide specific details as to which raw blot/gel images, if any, are not available. Email us at plosone@plos.org if you have any questions.

**Author response 3**: We have provided the original, uncropped, and unadjusted blot/gel images as required. All underlying image data are included in the Supporting Information files submitted with our revised manuscript.

4. PLOS requires an ORCID iD for the corresponding author in Editorial Manager on papers submitted after December 6th, 2016. Please ensure that you have an ORCID iD and that it is validated in Editorial Manager. To do this, go to ‘Update my Information’ (in the upper left-hand corner of the main menu), and click on the Fetch/Validate link next to the ORCID field. This will take you to the ORCID site and allow you to create a new iD or authenticate a pre-existing iD in Editorial Manager.

**Author response 4**: We confirm that the corresponding author's ORCID iD has been registered and successfully validated in the Editorial Manager system.

5. Please make sure that the Title in your manuscript file and the title provided in your online submission form are written in English.

**Author response 5**: We have carefully reviewed both the title in the manuscript file and the submission form, and confirm that they are now consistently written in English.

6. Please amend the title either on the online submission form or in your so that they are identical.

**Author response 6**: We have updated the title in the manuscript file to ensure it is now identical to the title provided in the online submission form. Thank you for your reminder.

Reviewer 1: Luteolin Alleviates CUMS-Induced Depressive-like Behavioral Deficits in Mice Through Blocking the JAK2/STAT3 Pathway

This article presents an interesting focus on the natural flavonoid luteolin (LUT) for the treatment of depression. The authors conducted an impressive battery of behavioral tests that supported their hypothesis, as well as some biological inflammatory tests.

I was impressed by the behavioral results, and I recommend publishing this article if the authors can address all the comments below:

Major comments:

1. Introduction: There is a lack of information about luteolin, particularly regarding the herbs that contain this flavonoid. How can luteolin influence the monoamine system, specifically the serotonin system? Are there any side effects associated with its use? Additionally, which companies offer luteolin for purchase?

**Author response 1**: Thank you for your question. Luteolin belongs to the flavone group of flavonoids and is widely distributed in members of the plant kingdom, such as Cirsium japonicum, Taraxacum officinale, Artemisia montana, and Cynara scolymus. This flavonoid is a polyphenol that has been widely used as a traditional medicine in China. Luteolin possesses hydroxyl groups at its 3′, 4′, 5, and 7 positions and a 2–3 carbon double bond, and its diverse biochemical and pathological activities are attributed to these structural characteristics. The activities of luteolin against diabetes, diabetic complications, and AD have been previously established. Similarly, luteolin also mediates the antidepressant-like effects of Cirsium japonicum extract, probably through potentiation of the GABAA receptor Cl– ion channel complex^[1]^. Likewise, in silico screening of phytochemicals from African medicinal plants identified luteolin, and its high docking score against hMAO-A (−98.5797 kcal/mol binding energy) suggested luteolin as an effective inhibitor^[2]^. A study of the effects of quercetin-related flavonoids and tea catechins on the MAO-A reaction in mouse brain mitochondria showed luteolin to be a superior inhibitor to quercetin^[3]^. Likewise, it also showed the potent inhibitory effect of luteolin on hMAO-A^[4]^. It suggests its potential therapeutic value in the treatment of neurodegenerative diseases. Luteolin is generally considered safe when consumed in dietary amounts, with common natural sources including vegetables such as celery, broccoli, and chrysanthemum. However, when taken in high doses as a supplement, it may cause mild gastrointestinal discomfort, such as nausea or abdominal bloating. Please refer to the content in section 1.2 “LUT (CAS No.: 491-70-3) was procured from Taoshu Biotechnology Co (purity > 99%).”.

2. Since luteolin is anticipated to have effects on treating depression, it would have been beneficial to include a positive control group that received an SSRI drug such as escitalopram or fluoxetine. However, since this control group was not included, it would be beneficial to explain why it was not included and to mention it as a limitation in the article.

**Author response 2**: Sincere thanks to the reviewer for the valuable suggestion regarding the addition of a positive control, such as fluoxetine. We fully understand the importance of a positive control for validating the experimental paradigm and providing a comparison with established antidepressant treatments. The primary aim of this study was to investigate whether luteolin alleviates depression-like behavior deficits induced by CUMS in mice by blocking the JAK2/STAT3 pathway, rather than to evaluate drug efficacy per se. Nonetheless, we highly value the reviewer's input. We will address this point in the limitations section of the manuscript and explicitly state that including a positive control will be a priority in our future follow-up studies to further confirm and expand upon the findings of this research.

3. Animals began receiving treatments after the first day of the chronic unpredictable mild stress (CUMS) model, indicating that they had not yet displayed any symptoms of anxiety or depression. Under these circumstances, the treatment serves as a preventive measure rather than a remedy for existing anxiety and depression. Therefore, the point of view of the article needs to be changed. In addition, the effective treatment received was at a high dose of luteolin (40 mg/kg), which may be because the high dose of luteolin in this treatment stabilizes this group in the baseline stage and does not allow it to develop the desired effects of depression and anxiety. It needs to be discussed in this article.

**Author response 3**: Thank you for your question. In this study, luteolin was administered after the successful establishment of the CUMS model, at which point anxiety- and depression-like behaviors had already manifested. Therefore, the intervention indeed represents a therapeutic treatment.

4. The study primarily examines the JAK2/STAT3 pathway and its relationship with inflammatory cytokines. However, it fails to clarify the link between blood and brain pathways, specifically how changes in the JAK2/STAT3 signaling in the brain affect blood cytokine levels. Additionally, the role of the hippocampus—selected as the focus area—remains unclear in terms of how it regulates cytokine levels in the blood.

**Author response 4**: This study does indeed have the limitations you pointed out. Our focus was primarily on the JAK2/STAT3 pathway and local inflammatory responses within the central nervous system, and we did not sufficiently elucidate the bidirectional regulatory mechanisms between the hippocampus and the peripheral immune system—particularly how changes in brain signaling pathways specifically affect peripheral blood cytokine levels. The absence of this mechanistic insight indeed limits a comprehensive understanding of the neuro-immune interaction network. In follow-up studies, we plan to simultaneously monitor inflammatory dynamics in both cerebrospinal fluid and peripheral blood, combined with regulatory experiments targeting specific neuronal populations in the hippocampus, to further investigate the specific pathways of central-peripheral immune communication and more systematically clarify their overall role in disease pathogenesis and progression. We greatly appreciate your valuable feedback.

Minor comments:

1. The open field test is an anxiety and motor test and not a depression test. In the method section, the authors wrote, “The number of entries into this central zone was recorded as measures of depressive-like behavioral deficits.” This is incorrect and should be corrected.

**Author response 1**: We sincerely appreciate the reviewer’s valuable feedback. The point raised regarding the interpretation of behavioral indicators in the open field test (OFT) is entirely valid. We have corrected the Methodology section to clearly state that the time spent in the central area is used to assess anxiety-like behavior rather than depression-like behavior. Thank you once again for helping us enhance the accuracy and scientific rigor of our manuscript.

2. Although this study uses inbred mice (C57/BL), eight mice per group is a relatively low number of animals, particularly in behavioral tests; it would be helpful to clarify why this low number of animals is used.

**Author response 2**: We thank the reviewer for the valuable comments on sample-size determination. We fully recognize that adequate sample size is critical to the reliability of our findings. During the experimental-design phase, we referenced comparable studies in the literature and confirmed that eight animals per group would satisfy the statistical requirements^[5]^.

3. At the FST and TST tests, it should have ignored the first two minutes and calculated the immobility time of the last four minutes. Maybe it was done during this article, but it is not mentioned.

**Author response 3**: We sincerely thank the reviewer for raising this critical technical point. Your suggestion regarding the forced swim test (FST) and tail suspension test (TST)—that an initial acclimation period should be excluded and immobility time calculated only during a specific subsequent interval—is entirely correct. We have added a clarification in the Methodology section (Section 1.3.4 and 1.3.5) of the manuscript stating that, in our experiments, the first 4 minutes were considered the acclimation phase and were excluded from analysis, while the total immobility time during the final 6 minutes was accurately recorded and analyzed. This approach allows for a more precise assessment of behavioral despair in mice. We greatly appreciate your thorough review, which has significantly enhanced the clarity and reproducibility of our experimental methods.

4. Depression and anxiety are more commonly observed in females than in males; however, this study was conducted using male mice, which should be clarified.

**Author response 4**: We sincerely thank the reviewer for raising this critical point. The issue of sex differences is indeed highly important. We have clarified this aspect in Section 1.1 (Animals) of the manuscript, explaining that male mice were used in this study primarily to avoid potential confounding effects of hormonal fluctuations caused by the estrous cycle in female mice on emotional behavioral tests.

5. The article focuses on proteins p-STAT3, STAT3, p-JAK2, and JAK2, but the connection between them is not so clear. It would be beneficial to explain the function of each protein and its relative expression in the results.

**Author response 5**: Thank you for your valuable feedback. We have revised and improved the relevant content in Section 2.5 (Results) of the manuscript, with additional elaboration on the functions of key proteins in the JAK2/STAT3 signaling pathway and their phosphorylation-mediated activation relationships. We kindly invite you to review these updates.

6. The western blot results should include all photos and not just the relevant cut of the proteins.

**Author response 6**: We thank the reviewer for the comment. The complete, uncropped original blot data are available and will be provided upon request.

7. This article focuses on treatment for depression but ignores anxiety disorders. Nevertheless, the article measured anxiety-like behavior using the EPM test and the Open field test but failed to mention this during the discussion. It is essential to include anxiety in the discussion to provide a more comprehensive understanding.

**Author response 7**: Thank you for your valuable feedback. We fully agree with your perspective that anxiety, as a frequently comorbid disorder with depression, warrants thorough discussion. We have now supplemented the Discussion section with an analysis of the EPM and OFT results, highlighting the potential role of luteolin in alleviating anxiety-like behaviors. Furthermore, we have integrated this effect with its antidepressant actions within a comprehensive mechanistic framework to provide a more holistic pathophysiological perspective. We kindly invite you to review these revisions.

**Reviewer2:**

The authors have shown that CUMS induces depressive-like behavior and increases p-JAK2, p-STAT3, and cytokine levels in mice. Furthermore, they’ve shown that luteolin reduces CUMS-induced effects on these parameters. My recommendation for this manuscript is to suggest major revisions for clarity and accurate representation of the statistics.

1.The statistical significance portrayed in the figures and legends does not match what is implied in the text. The figure captions state that the asterisks used on the LUT groups in the graphs show the significance compared to the control (no CUMS) group, but the data described in the text implies that the significance is in comparison to the CUMS group. Similarly, the figure captions state that the hashtag/number symbol was used to compare to the CUMS group, but that symbol is used on the CUMS group on the graph implying that the group was compared to itself. Either the statistics need to be described more clearly in the text or the figure captions just need to be updated.

**Author response 1**: Thank you for highlighting the important inconsistency between the figure annotations and the text description; it can indeed be confusing. In the revised manuscript we have clearly corrected this, ensuring that the * and # in the figure now correspond exactly to the statistical comparisons described in the text. We apologize for this oversight.

2.Did the authors complete a power analysis to determine how many animals should be used in the behavioral tests? Typically, 10-12 animals would be used for these behavioral tests so eight seems low. If a power analysis was performed, that information should be included in the Materials and Methods section.

**Author response 2**: We thank the reviewer for the valuable comments on sample-size determination. We fully recognize that adequate sample size is critical to the reliability of our findings. During the experimental-design phase, we referenced comparable studies in the literature and confirmed that eight animals per group would satisfy the statistical requirements^[5]^.

3.There is a discrepancy in the number of animals in the control group. The Materials and Methods section states n=6 but the results section states n=8.

**Author response 3**: Thank you for the reviewer's careful attention to the discrepancy in the number of animals in the control group. We confirm that this inconsistency was an oversight in the initial draft and has now been strictly corrected. In the Materials and Methods section, the sample size of the control group has been revised from n = 6 to n = 8 (specific location: 1.3.1 Model Preparation). We have rechecked the original experimental records and confirmed that the control group actually included 8 animals, and all experimental procedures were conducted based on this sample size. The earlier discrepancy was merely a clerical error during manuscript preparation and does not affect the authenticity of the data or the reliability of the conclusions.

4.Time spent in the central zone of the OFT was used as a measure of depressive-like behavioral deficits, and the results section states the OFT was used as a measure of motor abnormalities observed in depressed individuals. Citations should be included to show that the open field test has been used and validated in this way. The OFT is typically used to assess locomotor activity to ensure that a change in locomotor activity is not confounding the results of behavioral tests for depression, such as FST and TST, and not as a measure of depressive-like behavior itself.

**Author response 4**: Thank you for raising this important point. You are absolutely correct regarding the conventional primary use of the OFT, which is to assess baseline locomotor activity and rule out its confounding effects on tests like the FST/TST. In our study, while this was indeed a primary objective, we also referred to certain research precedents that utilize time spent in the center zone as an auxiliary indicator for exploratory behavior and lack of motivation^[6]^. We have revised the manuscript accordingly to clarify this rationale. We sincerely appreciate your thorough review.

5.The EPM is noted as being “commonly adopted to assess depressive-like behavioral deficits in rodents” and reference 7 is cited. Reference 7 does not use or state that EPM should be used as a measure of depressive-like behavior. Rather the EPM is used as a measure of anxiety. Because the results section described the EPM findings as anxiety, this may be a typo that can easily be fixed. If not, other references need to be included to show that the EPM has been used and validated in this way.

**Author response 5**: Sincere thanks to the reviewer for pointing this out. We have revised all relevant descriptions of the EPM from "depression-like behavior" to "anxiety-like behavior" testing (Section 1.3.3 Elevated Plus Maze) in accordance with the feedback. This was an oversight, and we appreciate your keen observation.

6.A positive control, *e.g.* fluoxetine, would be a welcome addition to this manuscript to validate the sensitivity of the paradigm and provide a comparison to established antidepressant treatments, however, it is not necessary for publication.

**Author response 6**: Sincere thanks to the reviewer for the valuable suggestion regarding the addition of a positive control, such as fluoxetine. We fully understand the importance of a positive control for validating the experimental paradigm and providing a comparison with established antidepressant treatments. The primary aim of this study was to investigate whether luteolin alleviates depression-like behavior deficits induced by CUMS in mice by blocking the JAK2/STAT3 pathway, rather than to evaluate drug efficacy per se. Nonetheless, we highly value the reviewer's input. We will address this point in the limitations section of the manuscript and explicitly state that including a positive control will be a priority in our future follow-up studies to further confirm and expand upon the findings of this research.

7.The results section mentions that “successful” CUMS mice were randomized into groups, but there is no mention of how “success” was defined. This criteria should be included in the Materials and Methods section.

**Author response 7**: Thank you very much for your valuable feedback. Your point regarding the need to clearly define the "success" criteria for the CUMS model is crucial for ensuring the reproducibility and scientific rigor of our study. In response, we have made the following additions and refinements to the Materials and Methods section in the revised manuscript, specifically within the "[1.3.1 Model Preparation]" subsection, by including the criteria that mice must meet to be considered as having successfully established the CUMS model.

8.The experimental set-up needs some clarification between the Materials and Methods section, the experimental workflow (Figure 1-1B), and the results section regarding the 2% ethanol solution (mentioned only in the results), 0.1 mL/kg of deionized water (mentioned only in the Materials and Methods), and DMSO (mentioned only in Figure 1-1B).

**Author response 8**: Thank you for your question. Based on the reviewers' comments, we have revised the descriptions of the solvents used in each group (including 2% ethanol solution and 0.1 mL/10g deionized water) in the relevant sections of the manuscript (Materials and Methods, and Results) to ensure consistency and clarity throughout the manuscript.

9.The results section should more transparently state the results of the low, medium, and high LUT groups. The results of the three LUT groups are often lumped together in the results section - stating that “LUT” had an effect – without mention of which dose(s) of LUT were significantly different in for each particular experiment.

**Author response 9**: We sincerely thank the reviewer for their valuable feedback. We have supplemented and revised the Results section to explicitly indicate which specific dose groups showed statistically significant differences in each experiment, thereby avoiding the generalized descriptions present in the original version.

10.The figure legends should state the type of tissue that was used for the experiments.

**Author response 10**: We sincerely thank the reviewer for this valuable suggestion. We have revised the figure legends accordingly to clearly indicate the type of tissue used in each experiment.

11.In section 2.2 of the results, it is stated in regard to the percentage of time spent in the open arms, “Although the other LUT doses showed a trend toward improvement, the differences were not statistically significant.” However, the graph shows that the LUT-M group was significant. The figure or text should be updated to reflect whichever information is correct. In addition, the p values have been stated, so mentioning a “trend” toward significance is irrelevant and unnecessary.

**Author response 10**: We sincerely thank the reviewer for their keen observation and valuable comments. You are absolutely right that the description of the "percentage of time spent with arms open" in Section 2.2 of the Results was inconsistent with the data presented in the figure. Following your suggestion, we have removed the unnecessary statements regarding "trends" and now directly and accurately report the statistical results. The current description is fully consistent with the information presented in the figure.

12.There is a typo in section 2.1 of the results section where “evtluate” is used instead of “evaluate”.

**Author response 12**: Thank you for your thorough review and for identifying the typographical errors in the text. We have corrected these in the revised manuscript and have conducted a comprehensive check of the entire document to ensure spelling accuracy. We sincerely apologize for any inconvenience caused and greatly appreciate your help in improving the quality of our manuscript.

Additional Editor Comments:

1. The purity of the studied compound should be specified in the Methods section.

**Author response 1**: Thank you for your valuable comment. We have added the purity information of the compound used in the **Section 1.2 Drugs** of the manuscript to ensure the accuracy and reproducibility of the methods. We kindly invite you to review this revision.

2. For statistical analysis of multiple group assays, a post hoc test should be applied following ANOVA.

**Author response 2**: Thank you for your valuable feedback. We have supplemented the description in the statistical analysis section of the manuscript to ensure the reliability of the statistical results. The specific content has been updated in the **Section 1.3.9 Statistical Methods** of the Methods chapter. We kindly invite you to review these revisions.

3. The novelty and innovative potential of your manuscript, compared to the published literature, should be described in detail in the abstract and discussion sections.

**Author response 3**: Thank you for your important suggestion. We have now elaborated in detail on the novelty and potential impact of this study compared to the existing literature in both the abstract and discussion sections.

[1]	de la Pena J B I, Kim C A, Lee H L, et al. Luteolin mediates the antidepressant-like effects of

in mice, possibly through modulation of the GABA

receptor [J]. Arch Pharm Res, 2014, 37(2): 263-9.

[2]	Zaib N, Naim A, Naeem S. Exploration of phytochemicals for inhibition of monoamine oxidase-A induced cancer using molecular docking studies [J]. Pak J Pharm Sci, 2019, 32(4): 1829-34.

[3]	Bandaruk Y, Mukai R, Kawamura T, et al. Evaluation of the Inhibitory Effects of Quercetin-Related Flavonoids and Tea Catechins on the Monoamine Oxidase-A Reaction in Mouse Brain Mitochondria [J]. J Agr Food Chem, 2012, 60(41): 10270-7.

[4]	Chimenti F, Fioravanti R, Bolasco A, et al. A new series of flavones, thioflavones, and flavanones as selective monoamine oxidase-B inhibitors [J]. Bioorgan Med Chem, 2010, 18(3): 1273-9.

[5]	Zhang M, Yu J, Liu A, et al. Luteolin in the Qi Bi Anshen decoction improves propionic acid-induced autism-like behavior in rats by inhibiting LRP1/MMP9 [J]. Phytomedicine, 2023, 118: 154965.

[6]	Dang R, Wang M, Li X, et al. Edaravone ameliorates depressive and anxiety-like behaviors via Sirt1/Nrf2/HO-1/Gpx4 pathway [J]. J Neuroinflammation, 2022, 19(1): 41.
